# Olfactory function testing before and after anesthesia

**DOI:** 10.1038/s41598-021-03400-x

**Published:** 2021-12-13

**Authors:** Anna Kristina Hernandez, Patrick Fuchss, Antje Haehner, Thomas Hummel

**Affiliations:** Present Address: Smell & Taste Clinic, Department of Otorhinolaryngology, TU Dresden, Dresden, Germany

**Keywords:** Drug therapy, Surgery

## Abstract

This study aimed to determine whether anesthesia would affect olfactory function. Patients who were admitted for surgical intervention that did not include the nasal cavity and paranasal sinuses were included in this prospective cohort study. Structured medical history was taken from the patients, including the following: age, sex, smoking history, alcohol intake, current medications, and sleep deficits prior to surgery. Before surgery, patients were asked for a self-rating of their olfactory function. Olfactory function was also measured using Sniffin’ Sticks comprising measures of odor threshold, discrimination, and identification. The mean interval between olfactory tests was 6 days (range 3–12 days). Seventy-three patients were included in the study, 34 men and 39 women. Olfactory scores were consistent before and after surgery as indicated by correlative analyses (*p* < 0.05). Odor thresholds, discrimination, identification, and composite TDI scores did not change significantly, whereas odor identification scores increased (*p* = 0.011) after surgery. In conclusion, post-operative olfactory scores remained stable. However, identification scores exhibited a slight increase which can be attributed to a retest effect. Overall, the present results indicate that surgery outside of the nasal and paranasal sinus region performed in general anesthesia has no major effect on the sense of smell.

## Introduction

Olfactory dysfunction occurs in approximately 5% of the general population^1,2^. Commonly reported etiologies include sinonasal conditions, viral infection, craniofacial trauma, neurodegenerative disorders, and drugs^2–6^. Postoperative changes to olfactory function are often observed following sinonasal^7–12^ and neurosurgery^13–17^, especially when changes occur in areas related to olfaction such as the olfactory bulb, cribriform plate, olfactory mucosa, or nasal septum. In the last 30 years, however, postoperative changes to olfaction have been also reported in other types of surgery, such as: cardiovascular^18,19^, abdominal^20–23^, and genito-urinary^24–26^.

There have also been several reports on effects of anesthesia^27^ on olfaction. Most reports involve general anesthesia^21–24,26,28–30^, however, there are a few citing spinal anesthesia^3,31^, intravenous^25^, and topical anesthesia^28,32,33^ to also have effects on olfaction. The rarity of such events may also mean that these cases are underreported.

The primary aim of the study was to determine the effect of anesthesia on olfactory function, among patients who underwent non-nasal surgery. To this end, patients should not only rate their sense of smell but olfactory function was measured in great detail to allow for relatively unbiased detection of possible subtle changes in olfactory sensitivity. Secondarily, we aimed to determine the relationship of the following variables: age, sex, smoking, alcohol intake, concomitant medication, pre-operative self-rating of olfactory function, preoperative sleep deficit, type of surgery, and duration of surgery to olfactory function score change after surgery among patients undergoing non-nasal surgeries.

## Materials and methods

This study was approved by the Institutional Review Board (IRB) of TU Dresden and was conducted according to the principles expressed in the Declaration of Helsinki. The study included a total of 73 patients (34 men and 39 women) of at least 18 years of age. Patients were admitted for surgical intervention at the Department of Orthopedics, Trauma and Reconstructive Surgery or the Department of Otorhinolaryngology at the University Clinic in Dresden. Inclusion and exclusion criteria can be found in Table 1.

**Table 1:** Inclusion and exclusion criteria.

Inclusion criteria	Exclusion criteria
At least 18 years old	Relevant previous ENT diseases (i.e., Chronic Rhinosinusitis, Allergic Rhinitis, Nasal Polyps)
Hospitalization for surgical treatment	Acute or pronounced chronic sinonasal inflammation
At least 3 days of hospitalization	Significant health impairment associated with olfactory disorders

Following a patient’s admission to the hospital, an explanation describing all possible risks and benefits related to participation in the study was given. Patients who gave their written informed consent to participate were interviewed. A structured medical history was taken^34^. This included the following details: age, sex, smoking history, alcohol intake, current medications, sleep deficits prior to surgery, and pre-operative olfactory self-rating.

Olfactory testing was done using the Sniffin’ Sticks test battery (Burghart Messtechnik, Wedel, Germany)^35^. Using commercially available felt-tip pens, a combination of olfactory threshold, odor discrimination, and odor identification score was determined.

For olfactory thresholds, a three-alternative forced-choice method was used. Three pens were held in alternating order at a distance of about two centimeters in front of both nostrils. Two of these pens contained an odorless solvent and one pen contained phenylethyl alcohol (PEA), a rose-like odor. The test contains 16 sets of three pens, each set with an increasing concentration of PEA. Set 16 has the lowest concentration of PEA, while set one has the highest. Participants were asked to select which pen among each triplet contained the odorant. Starting with the lowest odor concentration, a stepwise testing was done where two consecutive correct identifications of the odorous pen or one incorrect answer marked a “turning point”. This resulted to the presentation of the next triplet in decreasing or increasing concentration, respectively. The triplets were presented 20 to 30 s apart. The threshold score was defined as the average value of the last four turning points, with the final score ranging from 1 to 16. The participants were blindfolded to avoid visual identification of target pens. For odor discrimination, the same three-alternative forced choice method was used. Here, each set of three pens contained two pens with the same odorant and one pen with a different odorant. The participants were asked to identify the pen with the different odorant, in each of the 16 triplets. The score was the total number of correctly identified odorant pens, with a range of 0 to 16 points. Similar to the threshold subtest, participants were also blindfolded during the discrimination subtest to avoid visual identification of target pens. For odor identification, each participant was presented with 16 pens containing common and familiar odorants. Each participant must correctly identify and label each odorant using a list of four alternative descriptors. The descriptors were presented to the participants as labeled pictures. The interval between presentation of each pen was 20–30 s. The identification score was the sum of correctly identified odorant pens, and scores may range between 0 to 16 points. The composite TDI Score was the sum of scores for threshold, discrimination, and identification subtests, with a range of 1 to 48 points.

Participants were prohibited from eating, drinking anything other than water, and smoking one hour before the olfactory test. The first test was done the night before the surgery or on the morning before the scheduled surgery. Tests were usually done in the patients’ rooms, with careful consideration and measures to make the space as ideal as possible for testing (minimize other sources of odor/distractions or change study site as necessary). Shortly before patients were discharged, a second olfactory test was done under conditions comparable to the first test. In the case of multi-bed rooms, oral informed consent was obtained from all the other patients in the room prior to testing.

Patient records were assigned codes and anonymized. Data were encoded into a Microsoft Excel Office 365 version 2107 database (Microsoft Corp., Redmond, WA, USA) and checked for accuracy of encoding. Data analysis was done using SPSS ver. 28.0 (IBM, Chicago, IL, USA). Means and proportions were used to describe the study variables.

Pearson’s r was computed for age; Spearman’s rho for smoking, alcohol intake, pre-operative olfactory self-rating; independent two-tailed t-tests for sex and sleep deficits; paired two-tailed t-tests for pre- and post-operative TDI scores; repeated measures ANOVA for types of surgery and medications in relation to TDI scores, post-operative change in TDI scores; and with a *p* value of < 0.05 considered significant.

## Results

A total of 34 men and 39 women participated in the study, with ages ranging from 18 to 80 years old and a mean age of 51 years. The average olfactory testing interval was 6 days (SD 2.1, range 3–12 days). Most of the patients were non-smokers (n = 40, 55%), with occasional alcohol intake (n = 52, 71%), and without any current medications (n = 38, 52%). The majority of the individuals rated themselves to have normal olfaction (n = 48, 66%) and reported to have no sleep deficits prior to surgery (n = 48, 66%).

The types of surgeries the patients underwent are listed on Table 2. Most common surgeries done were hip endoprostheses (n = 17, 23%) and general hip surgery (n = 13, 18%). The mean duration of surgery was 76 min (SD 22.1, range 30 to 180 min) and the most common type of anesthesia used was general anesthesia (n = 62, 85%). Most people reported no change in olfactory self-ratings after surgery (n = 64, 88%).

**Table 2:** Frequencies, percentages and means.

Variables	Frequency	Mean (SD)
Age (in years)		51.01 (17.44)
**Sex**		
Male	34 (46.6%)	
Female	39 (53.4%)	
**Smoking**		
Never smoked	40 (54.8%)	
Stopped for > 1year	16 (21.9%)	
Smoker	17 (23.3%)	
**Alcohol intake**		
Never	17 (23.3%)	
Occasionally	52 (71.2%)	
Regularly	4 (5.5%)	
**Medications**		
No concomitant medication	38 (52.1%)	
Anti-hypertension	19 (26.0%)	
Nonsteroidal Anti-inflammatory Drugs (NSAIDs)	5 (6.8%)	
Oral Contraceptives	4 (5.5%)	
NSAIDs and Anti-hypertension	6 (8.2%)	
Others	1 (1.4%)	
**Pre-operative olfactory self-rating**		
Worse	9 (12.3%)	
Normal	48 (65.8%)	
Better	16 (21.9%)	
**Sleep deficit**		
No	48 (65.8%)	
Yes	25 (34.2%)	
**Operation**		
Foot	10 (13.7%)	
Hip	13 (17.8%)	
Knee	4 (5.5%)	
Spine	6 (8.2%)	
Upper Extremity	5 (6.8%)	
Hip Endoprosthesis	17 (23.3%)	
Knee Endoprosthesis	8 (11%)	
Ear	9 (12.3%)	
Throat	1 (1.4%)	
Duration of surgery (in minutes)		76.8 (22.1)
**Type of anesthesia**		
General	62 (84.9%)	
Spinal	1 (1.4%)	
Nerve block	10 (13.7%)	
Olfactory testing interval (in days)		6.1 (2.1)

### Pre- and post-operative change in composite TDI scores

Most post-operative changes to scores were observed in the threshold subtest, with less changes in the discrimination and identification subtests. Nine patients showed a change of at least 5.5 points on their post-operative TDI scores which is regarded as clinically significant^36^, four had worse TDI scores (“worse” group), while five had improved TDI scores (“better” group) (Table 3). All of the patients who experienced composite TDI score changes received general anesthesia (worse: n = 4, better: n = 5).

**Table 3:** Summary of changes in threshold, discrimination, identification, and composite TDI scores.

	Threshold^a^	Discrimination^b^	Identification^b^	Composite TDI^c^
Worse	11 (15.1%)	9 (12.2%)	1 (1.4%)	4 (5.5%)
Same	49 (67.1%)	59 (79.7%)	66 (90.4%)	64 (87.7%)
Better	13 (17.8%)	5 (6.8%)	6 (8.2%)	5 (6.9%)
Total	24 (32.9%)	14 (19%)	7 (9.6%)	9 (12.4%)

Scores were classified as “worse” or “better” when changes were greater or equal to the following: ^a^2.5 points, ^b^3 points, ^c^5.5 points^36^.

### Differences between patients improving and those worsening in function

**Figure 1:**
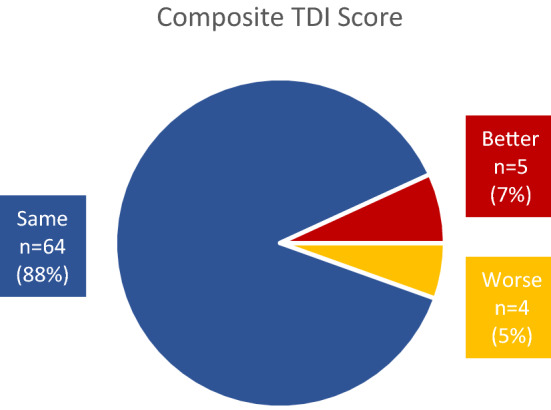
Pie chart of clinically significant changes in post-operative TDI Scores.

Mean age of those who had improved TDI scores was lower (age = 47 years) versus those whose scores worsened (age = 65 years). More women (n = 6) noted changes in their TDI scores than men (n = 3). However, only women (n = 4) had worse TDI scores, while men (n = 3) only had improved TDI scores (Fig. 1). The interval between testing was roughly the same between groups (range = 5–7 days). Hip endoprosthesis surgery had the most number of patients with changes in TDI score (worse: n = 2; better: n = 3). The worse and better groups were roughly the same in terms of smoking, alcohol intake, concurrent medication, and sleep deficit. Most of the patients that reported changes to TDI score were non-smokers (n = 8) with occasional alcohol intake (n = 7), and no concurrent medications (n = 6). Most individuals with changes in TDI score had no sleep deficits (n = 8). However, the group with changes to threshold scores had a larger number of participants with sleep deficits (worse: n = 4; better: n = 3). Mean duration of surgery was longer for patients with worse TDI score (duration = 88 min) versus those whose scores got better (duration = 70 min).

Patients had higher identification scores after surgery (t_72_ = 2.60, *p* = 0.011), with the mean post-operative identification score being 0.47 points higher than the mean of pre-operative scores (95% CI [0.109, 0.822]). Threshold, discrimination, and composite TDI scores had no significant difference before and after surgery.

### Secondary questions

Age was negatively correlated with changes in threshold score, but not discrimination, identification or composite TDI score. This means, the younger an individual, the more likely for threshold scores to improve post-operatively (rs_71_ = − 0.28, *p* = 0.018).

Sleep deficit had a significant effect on threshold scores, F_1,71_ = 8.953, *p* = 0.004. Patients without sleep deficits had significantly higher threshold scores pre- (t_72_ = 2.63, *p* = 0.011) and post-operatively (t_72_ = 2.57, *p* = 0.012).

Age was negatively correlated with pre-operative discrimination (r_71_ = − 0.46, *p* < 0.001), pre-operative identification (r_71_ = − 0.38, *p* < 0.001), and pre-operative composite TDI score (r_71_ = − 0.46, *p* < 0.001), but not pre-operative threshold score (r_71_ = − 0.19, *p* = 0.115).

Olfactory testing interval, duration of surgery, smoking, alcohol intake, and pre-operative self-rating of olfactory function were not correlated with changes in odor threshold, discrimination, identification, and composite TDI scores. Type of surgery or current medication had no effect on TDI scores.

## Discussion

There are only a few published articles about changes in olfaction related to anesthesia, and most of these are case reports or letters to editors. Some have attempted to determine the effect of anesthesia in controlled studies, however, only transient effects were noted at best^30,33^. There has been no clear causality or pathophysiology for these phenomena. Despite the presence of significant improvement in the identification scores among post-operative patients in this study, administration of anesthesia during surgery did not have a significant association with this change. We hypothesize that this observed change may simply be due to retest effect, which is defined as the increase in test scores due to the repeated administration of cognitive ability tests^37,38^. As odor identification testing is known to be affected by cognitive function, particularly executive processes and semantic memory^1,35,39^, we believe that retest effect may also apply to odor identification testing. An increase in odor identification scores with repeated testing has been previously reported in literature^40^.

General anesthesia suppresses neuronal signal conduction at various levels of sensory pathways, including olfaction. However, these effects are known to be transient and completely reversible^3,21^. A study by Kostopanagiotou et al.^29^ found that Sevoflourane, administered during general anesthesia, has transient effects on olfactory memory, and that this effect lasted up to 3 h after surgery. However, the interval for testing may have been too far apart (minimum of three days) to have measured any transient anesthesia effects.

Age was negatively correlated with clinically significant change in threshold score. This means the lower the age, the more likely for threshold scores to improve post-operatively. This was observed particularly more for women than men in this study. Cognitive function may play a role in this finding as retest effects were found to decline with age^41^. Younger participants may have benefitted from retest effects more. Conversely, another possible explanation is that older individuals may be more likely to experience cognitive decline. A study by Yahiaoui-Doktor et al.^42^ found an association between worsening of olfactory and cognitive performance.

Women have been found to have better olfactory performance, although the difference between sexes may be small and more observable in larger samples^35^. In our population, men had higher mean scores than women overall; but the significant difference was only observed in threshold scores. A meta-analysis by Sorokowski et al.^43^ found that women outperform men on threshold testing, but only by a small margin. Our comparably small sample size may be the reason why we did not observe a similar trend in our study. However, consistent with our findings, Sorokowski et al. also found that the effect size on threshold was twice as high as that of identification when it comes to differences in sex. Thus, threshold tests may be the most appropriate measure to check for potential differences in olfactory function among sexes^43^.

Sleep deprivation has been found to affect cognitive performance on a range of tasks^44–46^. Our findings were consistent with these as a significant decrease in pre- and post-operative threshold scores and pre-operative TDI scores were noted in patients with self-rated sleep deficits. Although discrimination and identification scores may be more associated with cognition, this may be in support of the idea that inadequate sleep can be associated with poor performance, especially in tasks that require attention and memory. Unfortunately, the amount of sleep deficit was not quantified in this study, as patients were only asked if they felt they have slept sufficiently long or otherwise. A more finite period of sleep deficit may be explored for future research.

Limitations to this study include the following: limited variability in the types of surgeries and anesthesia, low sample size, less than ideal sites of testing, and variable olfactory testing interval. Most were trauma surgeries, and limited information was gathered from other types of surgeries mentioned in literature such as cardiac and abdominal/gastrointestinal surgeries. Majority of our population received general anesthesia as well, and this may have led to biased results. As post-operative change in olfaction among non-nasal surgeries is an outcome that is relatively rarely reported in literature, a larger sample size may have better reflected the true presence or absence of this phenomenon. Although we attempted to best control the setting where the olfactory tests were done, it is unknown to what degree this had an effect to the patients’ scores.

Lastly, because this study was done before the pandemic, COVID-19 testing was not mandatory and COVID-19 associated olfactory loss was not considered as a variable in this study. However, possible effects of anesthesia on the sense of smell might be more pronounced in patients with pre-existing olfactory loss. Such situations can be expected to be slightly more frequent in the future as a consequence of the COVID-19 pandemic, with its ensuing olfactory loss^47^. Future studies may look at a larger sample, including patients with history of previous olfactory dysfunction (possibly those with previous COVID-19 infection), with standardized olfactory testing intervals to control for retest effects.

## Conclusion

Post-operative olfactory scores remain stable. However, identification scores exhibited a slight increase which can be attributed to a retest effect. Age was negatively correlated with change in threshold score, consistent with retest effects declining with age, as well as the association of olfactory and cognitive dysfunction with aging. Type of surgery or anesthesia had little or no effect on olfactory function. Overall, the present results indicate that surgery outside of the nasal and paranasal sinus region performed in general anesthesia has no major effect on the sense of smell.
